# Identification of biomarkers for immunotherapy response in prostate cancer and potential drugs to alleviate immunosuppression

**DOI:** 10.18632/aging.204115

**Published:** 2022-06-08

**Authors:** Jinpeng Zhang, Xiaohui Ding, Kun Peng, Zhankui Jia, Jinjian Yang

**Affiliations:** 1 Department of Urology, The First Affiliated Hospital of Zhengzhou University, Zheng Zhou University, Zhengzhou, Henan, China; 2 Henan Institute of Urology, Tumor Molecular Biology Key Laboratory of Zhengzhou, The First Affiliated Hospital of Zhengzhou University, Zheng Zhou University, Zhengzhou, Henan, China; 3 Department of Urology, Henan Province People’s Hospital, Zhengzhou University People’s Hospital, Zheng Zhou University, Zhengzhou, Henan, China

**Keywords:** prostate cancer, immune response, biomarkers, drug, immunotherapy

## Abstract

Background: Immunotherapy has a significant effect on the treatment of many tumor types. However, prostate cancers generally fail to show significant responses to immunotherapy owing to their immunosuppressive microenvironments. To sustain progress towards more effective immunotherapy for prostate cancer, comprehensive analyses of the genetic characteristics of the immune microenvironment and novel therapeutic strategies are required.

Methods: The transcriptome profiles of patients with prostate cancer were obtained from GEO and processed with the TIDE algorithm to predict their responses to immunotherapy. Next, the significant differentially expressed genes (DEGs) between the responder and non-responder groups were identified and used to compute the co-expression modules by WGCNA. Then, co-expression networks were constructed and survival analysis was applied to hub genes. Finally, drug candidates to alleviate immunosuppression were filtered in prostate cancer using GSEA based on hub genes.

Results: In total, we identified 2758 significant DEGs and constructed 16 co-expression modules, seven of which were significantly correlated with the immune response score. In total, 133 hub genes were identified, of which 13 were significantly associated with prostate cancer prognosis. Co-expression networks of hub genes were constructed with *KMT2B* at the center. Finally, six candidate drugs for prostate cancer immunotherapy were identified in PC3 and LNCaP cell lines.

Conclusions: We obtained datasets from multiple platforms, performed integrated bioinformatic analysis to identify 133 hub genes and 13 biomarkers of an immunotherapy response, and six candidate drugs were filtered to inhibit the immunosuppressive tumor microenvironment, to ultimately improve patient responses to immunotherapy in prostate cancer.

## INTRODUCTION

Prostate cancer (PRCA) is the most common cancer in men and is the secondary cause of cancer-related deaths in western countries according to the 2018 GLOBOCAN project [1]. Treatment options for localized PRCA include radical prostatectomy, radiation therapy, and androgen deprivation therapy (ADT). However, most patients undergoing ADT eventually progress to metastatic castration-resistant prostate cancer (mCRPC) [2]. In recent years, immunotherapy that stimulates the patient immune system to target cancer has emerged as a next-generation cancer treatment. Although immunotherapy has provided substantial benefits for many types of cancer, only a limited benefit was observed with mCRPC, owing to the dysfunctional immune system in PRCA, which promotes an immunosuppressive tumor microenvironment [3, 4].

**Figure 1:**
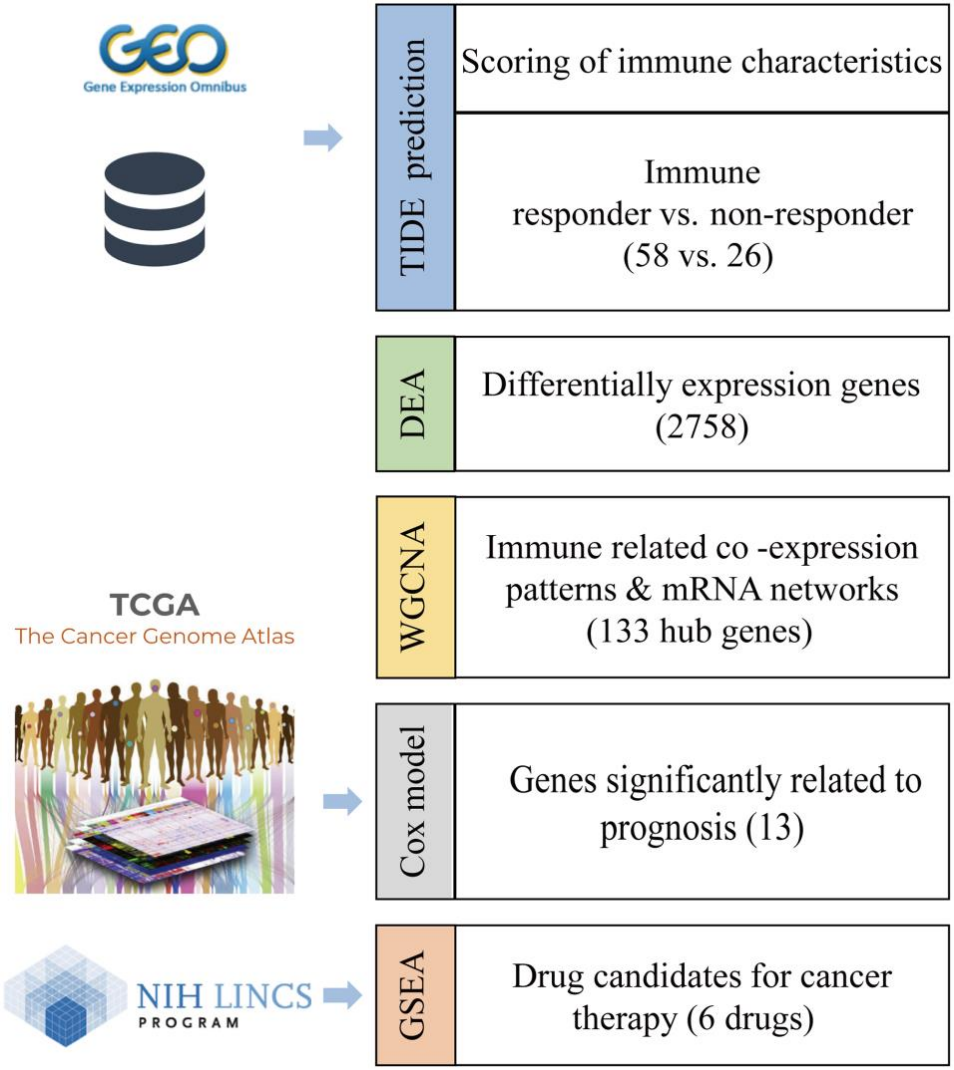
Workflow to identify the biomarkers of an immunotherapy response and candidate drugs.

Various therapeutic drugs have been used to enhance patient responses to immunotherapy. Targeting both CTLA-4 and PD-1 has been reported to result in a prostate-specific antigen response and objective response in some patients [5]. Moreover, to improve the efficacy of immunotherapy, researchers have focused on combination and sequential therapies [6]. For example, sipuleucel-T, an autologous cellular immunological agent, was the first immunotherapy approved by the FDA and has been shown to promote overall survival in patients with mCRPC [7]. Treatments using sipuleucel-T with anti-CTLA4 and anti-PD-L1 antibodies and an interleukin-15 (IL-15) superagonist are currently in clinical trials as new immunotherapy combination treatments [8]. In this study, we aimed to explore the genetic characteristics of the immunosuppressive tumor microenvironment and propose candidate drugs to improve patient responses to immunotherapy (Figure 1).

## RESULTS

### Prediction of immune response status by tumor immune dysfunction and exclusion (TIDE)

**Figure 2:**
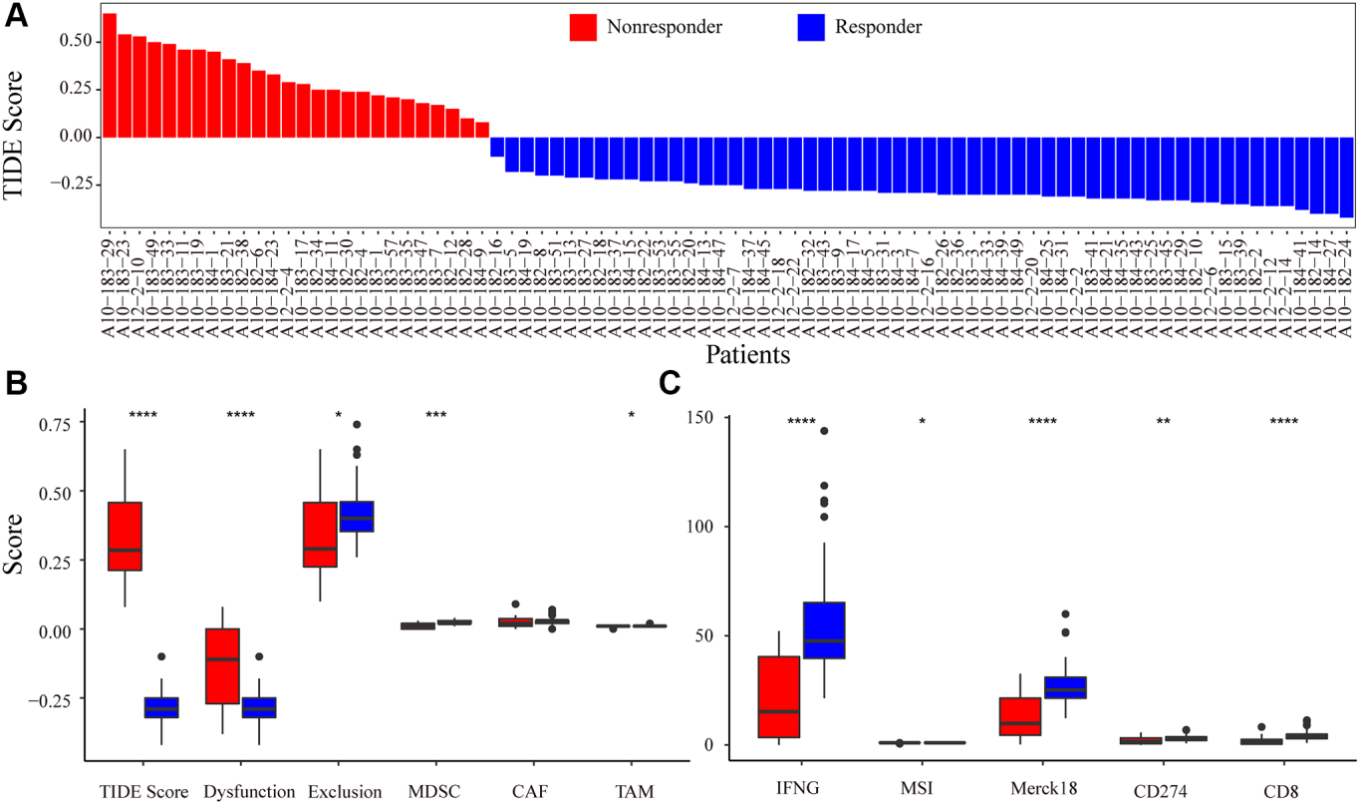
**Immune-response prediction by Tumor Immune Dysfunction and Exclusion (TIDE).** (**A**) Patients were predicted to be responders/non-responders to immunotherapy based on the TIDE score. (**B**) The score for immune features of TIDE score, Dysfunction, Exclusion, MDSC, CAF, and TAM predicted by TIDE. (**C**) The score for immune features of IFNG, MSI, Merck18, CD274, and CD8. Asterisks indicate the level of statistical significance: ^*^ < 0.05; ^**^ < 0.01; ^***^ < 0.001; and ^****^ < 0.0001.

The mRNA expression profile of PRCA patients was obtained from the Gene Expression Omnibus (GEO) database (GSE183019). We predicted the responses of 84 patients to immunotherapy and estimated the scores of 10 immune features with TIDE. Overall, 58 patients were predicted to be responsive to immunotherapy as their TIDE score was <0, whereas the other 26 patients were considered non-responders (Figure 2A). All 58 responder patients were CTL true (positive for five cytotoxic T lymphocyte markers, including CD8A, CD8B, GZMA, GZMB, and PRF1), indicating that the immune response to tumor T cell infiltration is highly consistent; that is, patients with high T cell infiltration have a high probability of responding to immunotherapy. Moreover, interferon gamma (IFNG) was a positive biomarker for the immune checkpoint blockade therapy response, and the score of IFNG in responder samples was significantly higher than that in non-responders. Except for the cancer-associated fibroblast (CAF) score, all immune feature scores showed significant differences between the groups. In addition, Merck18 (T-cell-inflamed signature), IFNG, CD8, and dysfunction signatures showed the most significant differences between the immune response and non-response groups (Figure 2B, 2C).

### Identification of immune response-related genes through differential expression analysis (DEA)

**Figure 3:**
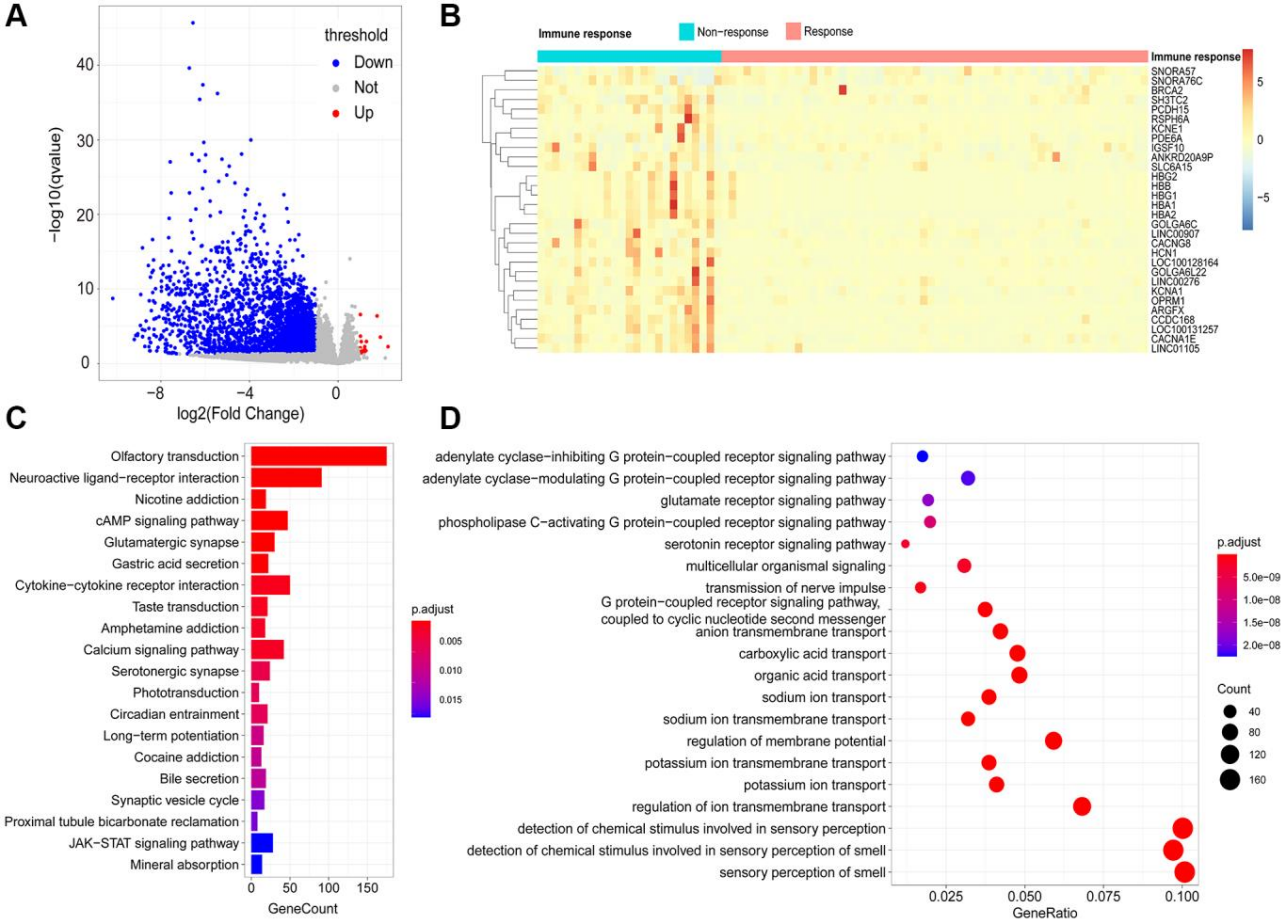
**Identification of immune-response related genes (IRRGs).** (**A**) Volcano plot of differentially expressed genes between responder and non-responder patient groups. In total, 2758 differentially expressed genes (DEGs) were considered as IRRGs. (**B**) Expression profiles of the top 30 significant IRRGs in responder and non-responder groups. The enriched KEGG pathways (**C**) and GO terms (**D**) of IRRGs were determined using ClusterProfiler.

By comparing the transcriptomes of 58 immune responder patients and 26 non-responder patients, 2758 immune response-related genes (IRRGs) were identified, and 99.42% (2742/2758) were expressed at low levels in immune-responsive samples (Figure 3A). The three most significant differentially expressed genes were *HBA2*, *LOC100131257*, and *CCDC168*. Notably, it has been reported that mutations in *CCDC168* are associated with adenosquamous carcinoma of the prostate [9]. The top 30 differentially expressed genes included multiple non-coding RNAs, such as *LINC00907*, *LINC01105*, *LINC00276*, *SNORA57*, and *SNORA76C* (Figure 3B); these non-coding RNAs can regulate gene expression through post-transcriptional modifications, thereby affecting the immune microenvironment of the prostate.

### Functional enrichment analysis of IRRGs

To explore the potential functional implications of IRRGs, we performed Gene Ontology (GO) and Kyoto Encyclopedia of Genes and Genomes (KEGG) enrichment analyses. As a result, the most significantly enriched pathways in KEGG were “olfactory transduction,” “cAMP signaling pathway,” and “calcium signaling pathway.” Moreover, “nicotine addiction” and “cytokine-cytokine receptor interaction” were also enriched in the KEGG pathways (Figure 3C). Similarly, GO enrichment analysis suggested that IRRGs were significantly enriched in material transport processes, such as “potassium ion transport,” “regulation of membrane transport,” and “anion transmembrane transport” (Figure 3D). Various signaling processes were also enriched, including “glutamate receptor signaling pathway” and “coupled receptor signaling pathway.” Functional annotation of IRRGs revealed that ion transport and signaling pathways are implicated in patient response to immunotherapy, thus contributing to anti-tumor immunosuppression.

### IRRG co-expression module identification with weighted gene co-expression network analysis (WGCNA)

In total, 2758 differentially expressed IRRGs were involved in the co-expression modules. First, the soft threshold was determined through the scale independence and mean connectivity analysis of modules with different power values ranging from 1 to 20 (Supplementary Figure 1). In this study, the power value (β) was set to 6 to produce a hierarchical clustering tree with different colors representing different modules. As a result, 16 total modules with different IRRGs were identified and displayed with different colors.

**Figure 4:**
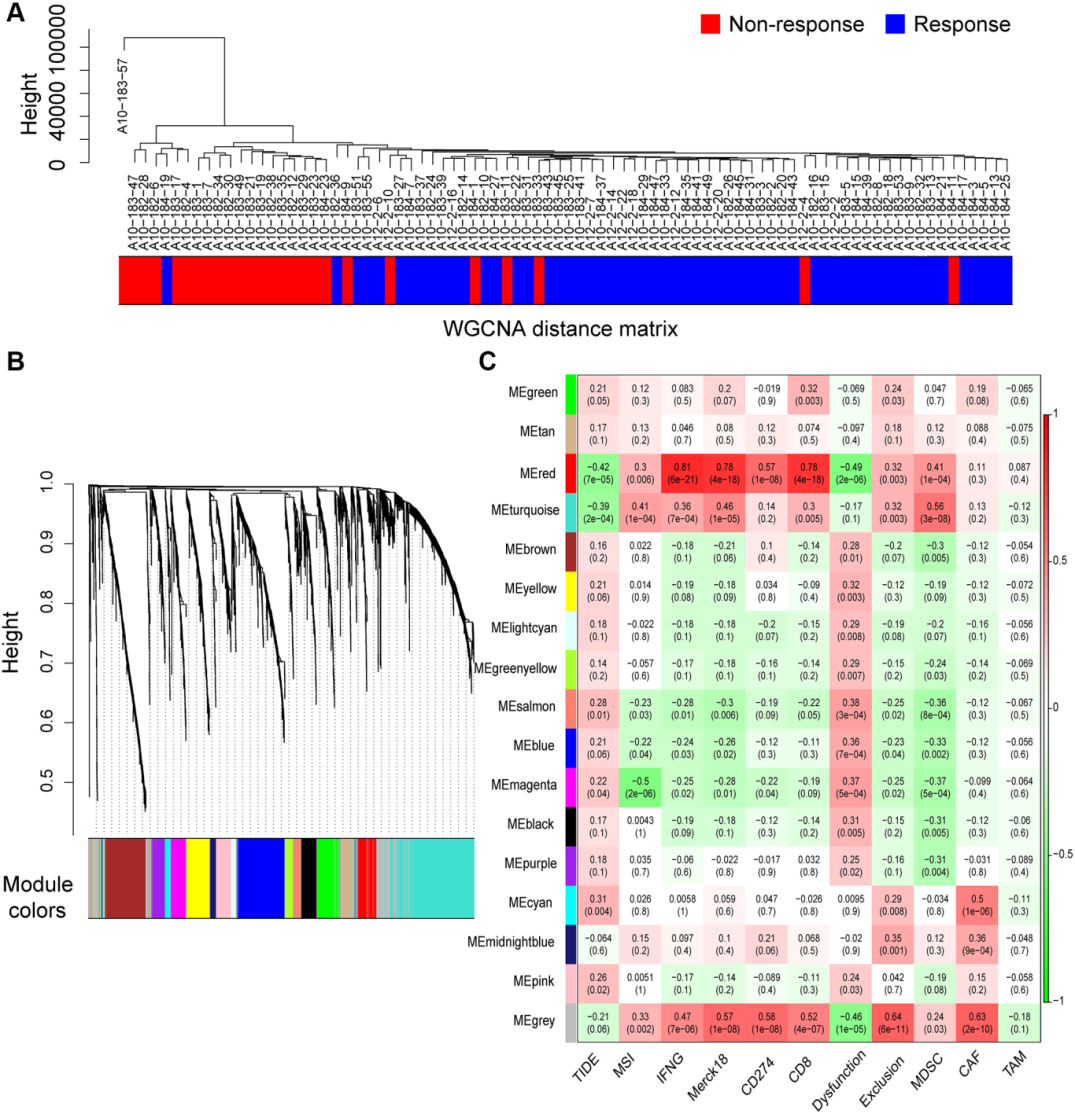
**Co-expression modules of immune-response related genes (IRRGs) identified by weighted gene co-expression network analysis (WGCNA).** (**A**) Sample dendrogram of patients based on transcriptome correlation. (**B**) Cluster dendrogram of IRRGs and 16 co-expression modules were identified by WGCNA. (**C**) Correlation coefficients between co-expression modules and immune features (above) with *p*-values (below).

The hierarchical clustering dendrogram of the patients based on the WGCNA distance matrix is shown in Figure 4A, and the immune responding patients were grouped together. IRRGs were grouped into 16 co-expression modules (Figure 4B), and the correlation between modules and immune features is displayed in Figure 4C, which suggested that turquoise module was significantly correlated with Merck18, IFNG, and myeloid-derived suppressor cells (MDSCs). Seven modules that were significantly associated with TIDE score, with *p*-value < 0.05, were retained for further analysis.

**Figure 5:**
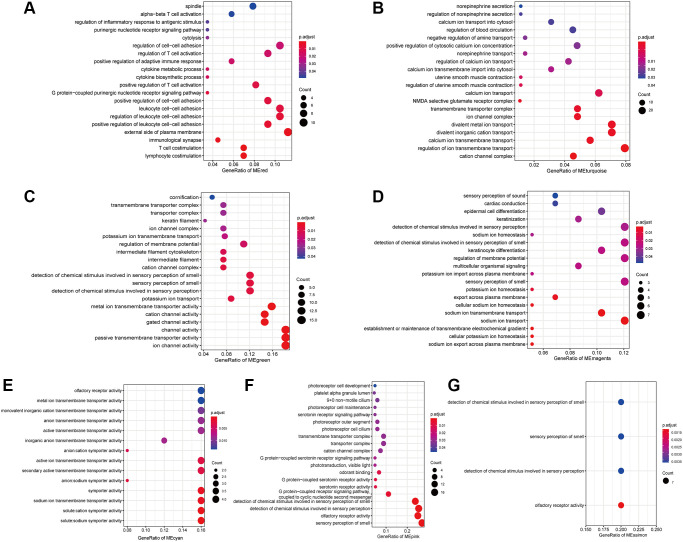
The enriched GO terms of IRRGs in the red module (**A**), turquoise module (**B**), green module (**C**), magenta module (**D**), cyan module (**E**), pink module (**F**), salmon module (**G**).

To provide insight into the biological functions of the IRRGs in seven immune response-related modules, we performed GO (Supplementary Table 1) and KEGG (Supplementary Table 2) enrichment analysis. Most modules included enriched GO terms related to voltage-gated channels, such as “cation channel complex” (3/7), “ion transport” (5/7), and “sensory perception of smell” (4/7). Specifically, the IRRGs involved in the red module were significantly enriched in immune-related GO terms, such as “lymphocyte/T cell costimulation” “, immunological synapse ” “, positive regulation of adaptive immune response”, and “cytokine metabolic process” (Figure 5A). IRRGs of turquoise module were enriched in the “regulation of uterine smooth muscle contraction” (Figure 5B). Further, IRRGs of green (Figure 5C), magenta (Figure 5D), and cyan (Figure 5E) modules were mostly enriched in pathways related to “ion transport” and those of magenta module were especially enriched in “epidermal cell differentiation”. IRRGs of pink (Figure 5F) and salmon (Figure 5G) module were mostly enriched in “sensory perception of smell” and “olfactory receptor activity”, respectively.

### Identification of biomarkers of immunotherapy responses

**Figure 6:**
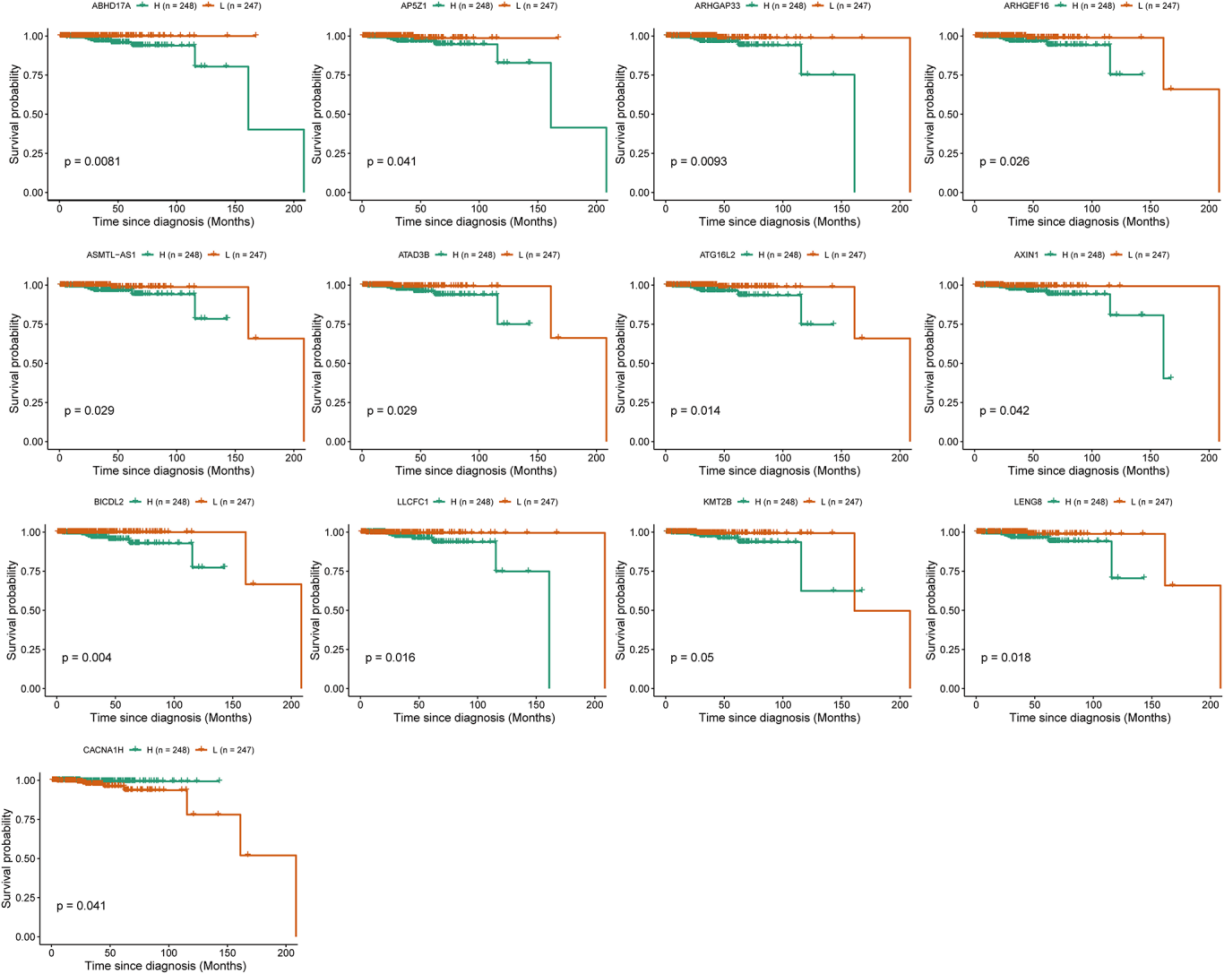
**Survival analysis identified 13 immune-response related genes (IRRGs) significantly related to the prognosis of prostate cancer (PRCA).** Patients were divided into a high expression group (H) and low expression group (L) according to the median expression of specific genes.

To investigate the role of IRRGs as potential marker genes in immunotherapy response, we first identified hub genes in seven modules of interest (see Methods). Ultimately, 133 genes were identified as hub genes, which were selected for survival analysis (Table 1), and 13 of them were found to be significantly associated with PRCA prognosis. *BICDL2*, a protein-coding gene predicted to enable small GTPase binding activity, showed the most significant association with PRCA prognosis (*p*-value = 0.004). Moreover, the rest of the prognosis-related genes were as follows: *ABHD17A* (*p*-value = 0.0083), *ARHGAP33* (*p*-value = 0.0093), *AP5Z1* (*p*-value = 0.041), *ARHGEF16* (*p*-value = 0.026), *ASMTL-AS1* (*p*-value = 0.029), *ATAD3B* (*p*-value = 0,029), *ATG16L2* (*p*-value = 0.014), *AXIN1* (*p*-value = 0.042), *LLCFC1* (*p*-value = 0.016), *LENG8* (*p*-value = 0.018), *KMT2B* (*p*-value = 0.05), and *CACNA1H* (*p*-value = 0.041) (Figure 6). The IRRGs described previously herein were considered biomarkers of immunotherapy response in PRCA, which might affect the immune microenvironment, thus playing an important role in patient responses to immunotherapy and determining clinical outcomes.

**Table 1:** Number of immune-response related genes (IRRGs) included in seven modules that are significantly correlated with the tumor immune dysfunction and exclusion (TIDE) score and the hub genes in each module.

**Module color**	**Number of IRRGs**	**Most-related immune feature**	**Hub genes (number of hub genes)**
Red	125	IFNG	*CDKN3* (1)
Turquoise	549	MDSC	*PAGR1*, *SIX5*, *PCED1A*, *BAHCC1*, *ARMC5*, *ATN1*, *SNRNP70*, *LOC100133091*, *KMT2B*, *CHPF*, *ABHD17A*, *CAPN15*, *SGSH*, *TMEM250*, *SAFB2*, *AMIGO3*, *ANO8*, *SDF4*, *H1FX*, *ARRDC1*, *SNAPC4*, *INPP5E*, *SLC12A9*, *ABHD15*, *SLC43A2*, *PCNX3*, *LLCFC1*, *PIM3*, *PPP1R3F*, *CASZ1*, *H1FX-AS1*, *PALM*, *SLC39A3*, *AGRN*, *GPR137*, *ARFGAP1*, *LENG8*, *ARHGAP33*, *BCAR1*, *CACNA1H*, *AGPAT2*, *CAPN10*, *SHC2*, *GUSBP11*, *GPANK1*, *SLC4A3*, *KIFC3*, *SCRIB*, *AP5Z1*, *ADCK2*, *AXIN1*, *ARHGEF1*, *GLTSCR1*, *INTS1*, *RNPEPL1*, *SH3TC1*, *ARFRP1*, *C19orf25*, *PARP10*, *CFAP410*, *ATG16L2*, *SNORD17*, *HDAC10*, *BICDL2*, *RRN3P3*, *C15orf39*, *ASMTL-AS1*, *ALDH16A1*, *ATAD3B*, *SCARF2*, *ATG4D*, *ARHGEF16*, *CLASRP*, *PPP1R16A*, *ANAPC2*, *CCDC9*, *BORCS6*, *PPP2R3B* (78)
Cyan	45	Dysfunction	*LINC01360*, *SLC13A1*, *ALPG*, *LINC02881*, *OR7E5P*, *IGFBP1*, *CA9*, *OR1E2* (8)
Salmon	60	Dysfunction	*LINC01104*, *OR4F17*, *LINC01304*, *OR6C1*, *PLSCR5*, *PDYN*, *OR5AC2* (7)
Pink	114	Dysfunction	*KIF4B*, *CD1A*, *ANKRD18DP*, *PIH1D3*, *SATB2*, *AS1*, *ACOD1*, *GFY*, *GK2*, *LINC01180*, *C1orf185*, *HCRTR2*, *OR5AK2*, *OR1G1*, *C1orf141*, *LINC00297*, *LINC00433*, *OR2V1*, *ROPN1L-AS1* (18)
Magenta	108	MSI	*POU4F2*, *LINC01159*, *SLC5A12*, KRTAP5-8, *CADM2-AS2*, *ACTR3BP2*, *CD5L*, *LOC100132735*, *CETN1* (9)
Green	151	CD8	*OR56B4*, *KRT38*, *KRT78*, *C14orf180*, *OR2B11*, *OR4D6*, *HMGA1P7*, *KRT39*, *KCNK18*, *OR5M9*, *C10orf120*, *PDHA2*, *KRT84* (13)

Abbreviations: IFNG: interferon gamma; MDSC: myeloid-derived suppressor cell; MSI: microsatellite instability.

### Construction of co-expression network

**Figure 7:**
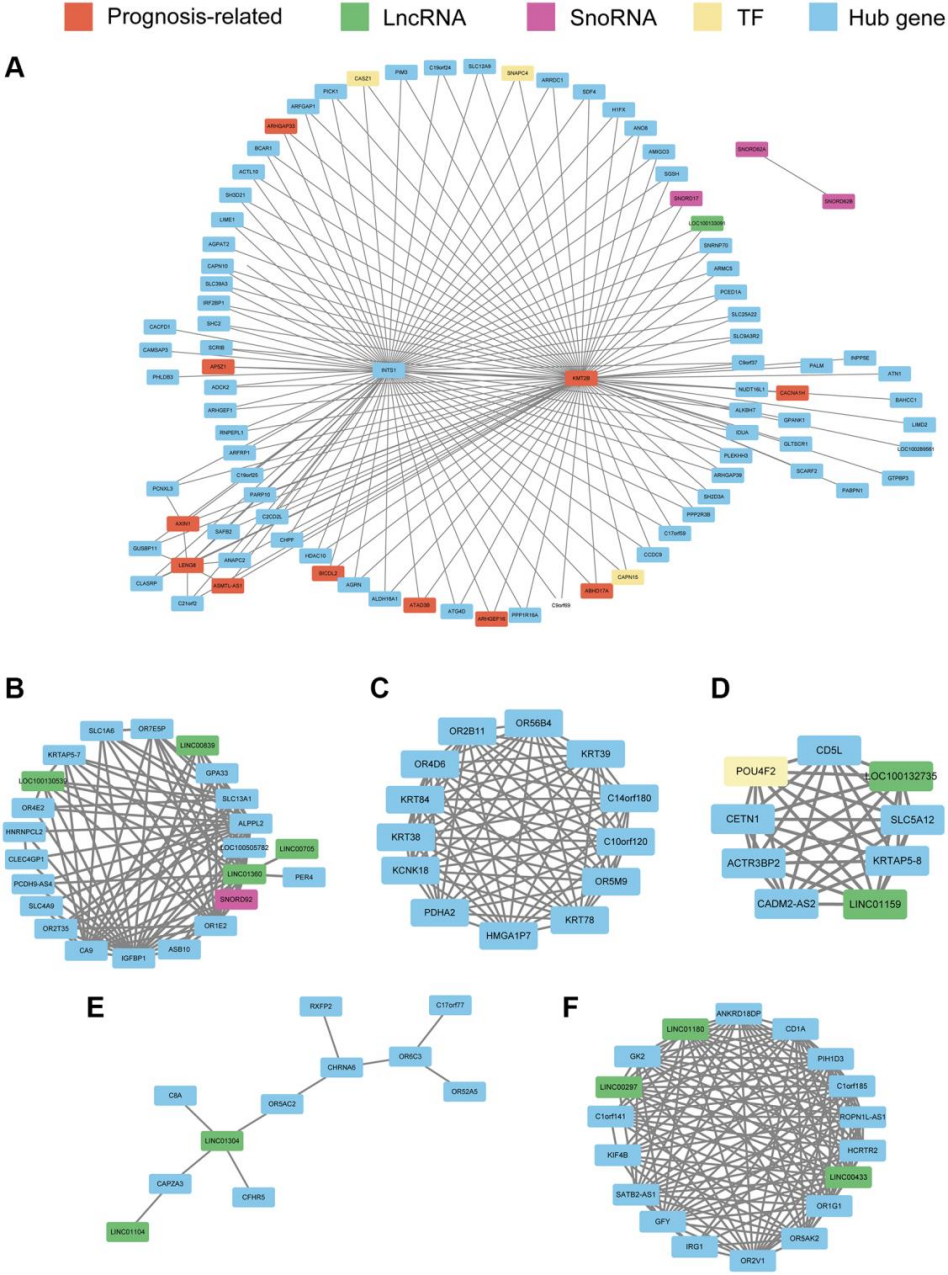
Co-expression network constructed with hub genes in the turquoise module (**A**), cyan module (**B**), green module (**C**), magenta module (**D**), salmon module (**E**), and pink module (**F**). Every node represents a hub gene or hub gene of co-expressed genes; genes significantly correlated with prognosis are colored red. LncRNAs, SnoRNAs, and transcription factors (TFs) are vital regulatory molecules and are colored with green, purple, and yellow, respectively. For the turquoise module, to obtain a clear picture, the edges weighted above 0.25 are displayed, whereas in the other six modules, edges weighted above 0.1 are displayed.

Using WGCNA, we constructed co-expression networks of the hub genes in the seven modules identified previously herein based on expression correlations to provide insights into the role of signature genes in PRCA pathogenesis. Excluding the red module, which had only one hub gene, we constructed networks for the other six modules. The results showed that in the turquoise module, *INTS1* and *KMT2B* were at the center of the network. *KMT2B* was significantly related to survival and was related to multiple regulators of gene expression (Figure 7A), such as *LOC100133091* and *SNORD17*, as well as the transcription factor (TF)-encoding genes *CAPN15*, *SNAPC4*, and *CASZ1*; these regulators themselves act as modular hub genes and might have regulatory functions. There were four lncRNAs and one snoRNA involved in the network of cyan module (Figure 7B). In green module, the co-expression relationship among 13 hub genes was presented (Figure 7C) and nine hub genes, including two lncRNAs and one TF-encoding gene were used in magenta module (Figure 7D). For hub genes in salmon and pink modules, the networks were constructed and presented in Figure 7E and 7F, respectively.

### Identification of small molecules targeting core IRRGs

Having identified PRCA signature genes significantly associated with the immunotherapy response, most of which were downregulated in immune-responsive patients, we hypothesized that when signature genes are targeted with small molecules, the downregulated expression of these genes would sensitize cells to the drug. Such molecules could potentially reverse tolerance and facilitate therapy effectiveness when combined with traditional drugs. Data from the Library of Integrated Network-Based Cellular Signatures (LINCS) database were used to identify small molecules targeting PRCA signature genes, with their co-expressed genes were identified by WGCNA.

**Figure 8:**
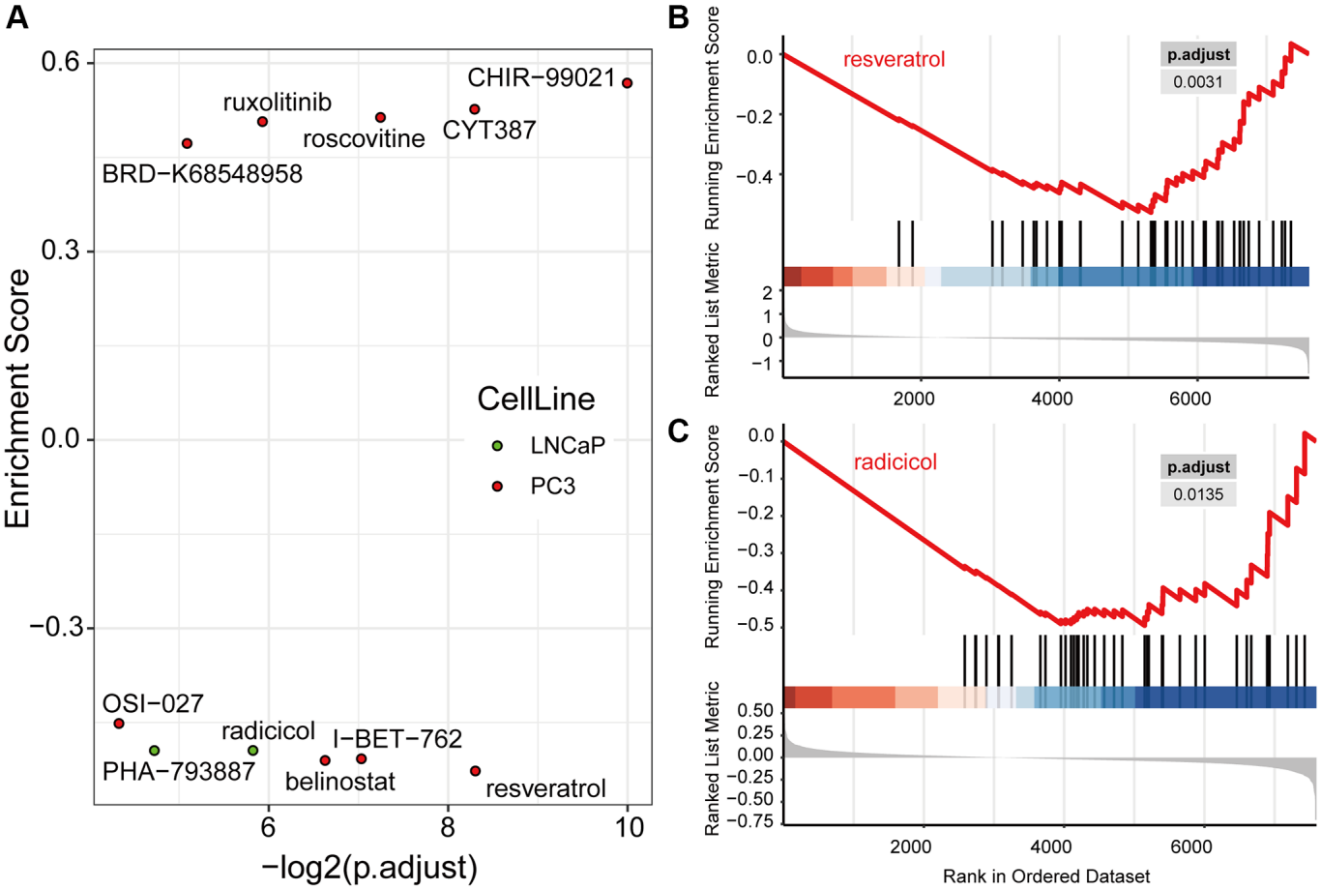
**Drug candidates were filtered from the LINCS database, which were predicted to contribute to immunotherapy.** (**A**) Nine drugs in PC3 and two drugs in LNCaP cells were significantly regulated hub genes computed by Gene Set Enrichment Analysis (GSEA). The GSEA plots of resveratrol (**B**) and radicicol (**C**), the most significantly effective drugs for the PC3 and LNCaP cell lines, respectively, show their regulation of hub genes.

Based on the signature genes and the co-expression pairs identified by WGCNA, from hundreds of small molecules, we identified nine and two candidate drugs, respectively, from the expression profiles of the PC3 and LNCaP cell lines from the LINCS database (Figure 8A, Supplementary Table 3). These drugs regulated the expression of signature genes of PRCA significantly. Among them, four and two compounds, respectively, were considered for immunotherapy based on PC3 and LNCaP cells, and these significantly inhibited the expression of signature genes, making them potential candidates for combination therapies. For PC3 cells, resveratrol most significantly inhibited the expression of signature genes (Figure 8B), whereas in the LNCaP cell line, radicicol (Figure 8C) and PHA-793887 had the most significant effects. No compound was observed to have significant effectiveness in both cell lines.

## DISCUSSION

In this study, PRCA patients were divided into immune-responder and non-responder groups using a computational method. Between immune responders and non-responders, 2758 IRRGs were identified and clustered into 16 co-expression modules, seven of which were significantly correlated with the TIDE score. From seven modules of interest, 133 total hub genes were identified. The red module had only one hub gene that met the criteria, cyclin-dependent kinase inhibitor (*CDKN3*), which was upregulated in the immune responder sample group. As a tumor-promoting gene, *CDKN3* encodes a protein that plays an important role in protein phosphorylation and cell cycle regulation [10]. Many studies have shown that alternative splicing and mutations in *CDKN3* are related to the cellular immune microenvironment in liver cancer, and experiments conducted by Huang et al. showed that high expression of *CDKN3* can be triggered by Tfh cell-derived signals, an epigenetic mechanism regulating activated B cells [11]. It is possible that *CDKN3* promotes the proliferation of immune cells while promoting the proliferation of tumor cells, so that samples with high expression levels of *CDKN3* are more likely to respond to immunotherapy.

Survival analysis of 133 hub genes identified 13 genes significantly associated with PRCA prognosis, specifically *CACNA1H*, *BICDL2*, *ABHD17A*, *ARHGAP33*, *AP5Z1*, *ARHGEF16*, *ASMTL-AS1*, *ATAD3B*, *ATG16L2*, *AXIN1*, *LLCFC1*, *LENG8*, and *KMT2B*, which were considered biomarkers for the immunosuppressive microenvironment. Low expression of these genes was associated with better prognosis, with the exception of *CACNA1H*, which encodes a protein involved in the voltage-dependent calcium channel complex; high expression of *CACNA1H* was found to be significantly associated with better prognosis, suggesting that ion transport plays an important role in PRCA. It has been reported that Cav3.2 T-type Ca^2+^ channels exist in 100% of PRCA patients, and Cav3.2 voltage-dependent calcium channels are involved in cell growth in PRCA [12]. Thus, *CACNA1H* might affect the response of patients to immunotherapy by affecting the function of ion channels. Further, researchers also found that somatic mutations in *CACNA1H* recur at the site of tumor metastasis, which could be related to immune escape in colorectal tumors [13]. *ABHD17A* (α/β-hydrolase domain-containing protein 17a) is another biomarker related to voltage channels. Previous research demonstrated that *ABHD17A* modulates ion channels at the post-transcriptional level by deacetylating the stress-regulated exon domains of large conductance voltage- and calcium-activated potassium channels [14]. Moreover, low expression of *ABHD17A* is associated with improved overall survival of PRCA according to survival analysis.

Previous studies have shown that some of the screened biomarkers affect the progression of various tumors through different molecular mechanisms. Regarding *ARHGAP33*, researchers have reported that it is a marker gene that can predict prognosis in prostate cancer [15], and *ARHGAP* family genes have been demonstrated to promote bladder cancer progression by establishing a tumor-promoting microenvironment [16]. *ARHGEF16*, encoding a nucleotide exchange factor that catalyzes the exchange of GDP nucleotides for GTP, is critical for cell proliferation, growth, and tumorigenesis in various cancers [17, 18]. Hiramoto-Yamaki et al. reported that ARHGEF16 could modulate the migration of breast cancer cells in a RhoG-dependent manner [19]. Further studies have shown that *ARHGEF16* activates RhoG and PI3K, contributing to apoptosis resistance in tumor cells [20], and interacts with *CKAP5* to promote the proliferation and migration of glioma cells [21]. Although the critical functions of ARHGEF16 have been reported in many cancer types, the underlying mechanism of its effect on PRCA prognosis remains to be elucidated. Moreover, *ASMTL-AS1*, a noncoding transcript, inhibits β-catenin expression and inactivates carcinogenic Wnt/β-catenin signaling in breast cancer [22]. *ATG16L2* plays an important role in T cell autophagy [23]. *AXIN1* contains a G-protein signaling regulation domain and a dishevelled and axin domain, and many studies have reported its modulation of immune-related signaling pathways in liver cancer [24, 25]. In addition to the genes previously mentioned, *BICDL2*, *LLCFC1*, *LENG8*, *KMT2B*, *AP5Z1*, and *ATAD3B* were also found to be IRRGs significantly associated with PRCA prognosis in this study, which were proposed to have vital roles in the response to immunotherapy in PRCA and require further study.

Based on the hub genes and co-expression pairs identified by WGCNA, we identified candidate drugs from hundreds of small molecules based on the mRNA expression profiles of the PC3 and LNCaP cell lines from the LINCS database. In PC3 cells, the most significantly effective drug, resveratrol, is a phytoestrogen with antioxidant, anti-inflammatory, cardioprotective, and anti-cancer properties [26]. Previous studies revealed that resveratrol can reverse multidrug resistance in cancer cells, and when used in combination with clinically used drugs, it can sensitize cancer cells to standard chemotherapeutic agents [27]. There have been many reports that resveratrol can inhibit tumor processes by regulating multiple pathways, and the pathways affected by resveratrol, such as the PI3K pathway, Wnt signaling, and inflammation-related pathways, affect the PRCA immunosuppressive microenvironment [28]; thus, resveratrol could become an adjuvant drug for immunotherapy to help improve its effectiveness. I-BET-762 is also well studied and was reported to reduce MYC expression in PRCA and subsequently inhibit cell growth and reduce the tumor burden [29]. Moreover, researchers have reported that bromodomain and extra-terminal (BET) bromodomain inhibition can mediate changes in expression at a genome-wide level in PRCA cells and increase the susceptibility of cancer cells to CD8 T cell targeting [30]. Thus, I-BET-762 acts as a BET inhibitor and could have clinical benefits for PRCXA patients in combination with immunotherapy. The other four proposed drugs—belinostat, OSI-027, radicicol, and PHA-793887—are less well-studied in relation to PRCA treatment. The FDA approved belinostat for the treatment of patients with relapsed or refractory peripheral T-cell lymphoma [31] and OSI-027 (also known as ASP7486) is a dual mTORC1/mTORC2 ATP-competitive kinase inhibitor [32]. Compared with the properties of PC3 cells, LNCaP cells might represent a striking feature of early androgen-dependent PRCA, as the significantly enriched drug radicicol potentiates radiation-induced cell killing in a hormone-sensitive PRCA cell line through degradation of the androgen receptor [33]. PHA-793887 is an inhibitor of multiple cyclin-dependent kinases (CDKs), with activity against CDK2, CDK1, and CDK4, and has been validated to enhance immunotherapy against melanoma [34]. However, few studies have investigated the effects of these four drugs on PRCA, and thus, further study is necessary.

Although the biomarker genes and candidate drugs that affect immunotherapy were mostly proven by existing studies, there are certain limitations to this study. The mRNA expression profile obtained from the LICNS database only includes the expression of 30.08% (40/133) hub genes, which possibly prevents the accurate prediction of candidate drugs (Supplementary Table 4). Moreover, the mechanisms underlying the effects of biomarkers require further investigation and experimental validation, as information on the ability of the proposed drugs to improve immunotherapy in PRCA is, apart from reported cases, lacking. Further research will focus on the mechanism underlying the effects of the proposed biomarkers, and more experiments are needed at the protein level and *in vivo*.

In conclusion, we integrated and analyzed datasets from multiple platforms to identify 133 hub genes in seven co-expression modules. Co-expression networks were constructed to reveal the regulatory relationships between them. Survival analysis identified 13 genes that were significantly associated with prognosis and considered biomarkers of the immunotherapy response for PRCA. Furthermore, we proposed six candidate drugs, two of which, resveratrol and I-BET-762, have been reported to be beneficial for PRCA treatment, whereas the other four, belinostat, OSI-027, radicicol, and PHA-793887, remain to be studied.

## METHODS

### Data source

The mRNA expression profiles and dataset comprising clinical information of PRCA patients of GSE183019 were acquired from the GEO (https://www.ncbi.nlm.nih.gov/geo/) database [35, 36] and The Cancer Genome Atlas (TCGA, https://portal.gdc.cancer.gov) database [37]. Excluding normal tissue samples, 84 PRCA samples with count profiles of 25498 expressed genes were retained for further analysis. The transcriptome profiles of the PC3 and LNCaP cell lines with different drug perturbations were obtained from the LINCS (https://lincsproject.org/LINCS/) database [38], and data of a total of 1121 compounds with the expression of 7163 genes were collected.

### Prediction of immune response status

TIDE [39] is an integrated web tool used to predict patient responses to immunotherapy. Based on tumor expression profiles, TIDE can score multiple transcriptomic biomarkers of several immune features, including immune system dysfunction, T cell exclusion, MDSCs, IFNG signature, CAFs, M2 subtype of tumor-associated macrophages (TAMs), microsatellite instability (MSI), Merck18, CD274, and CD8. These features were demonstrated to be related to patient responses to immunotherapy, and we calculated the estimated score of the immune features for each patient based on their mRNA expression profiles. Specifically, patients with a TIDE score < 0 were considered responders, whereas patients with a TIDE score > 0 were considered non-responders.

### Identification of immune response-related genes

Based on the immune response status predicted by TIDE, differentially expressed genes were calculated between responder and non-responder patient groups using DEA with the R package DESeq2 [40] in R software. The significantly differentially expressed genes were identified using the criteria of |log_2_ fold-change| > 1 and adjusted *p*-value < 0.05. Genes meeting the criteria were considered IRRGs.

### Functional annotation of immune response-related genes

To explore the potential biological functions and pathways associated with IRRGs, we performed GO (http://geneontology.org/) [41] and KEGG (https://www.genome.jp/kegg/) [42] pathway analysis using the R package “ClusterProfiler” [43]. GO terms and KEGG pathways with a *p*-value < 0.05 were considered significantly enriched.

### Identification of co-expression modules for immune-related genes

IRRGs were used to construct co-expression modules with the WGCNA [44] package in R software. Pearson’s correlation matrices were calculated between each pair of retained IRRGs from the corresponding expression levels. Clusters were obtained from the dendrogram by applying the dynamic tree-cutting technique [45]. Module-trait relationships were calculated according to the correlation between modules and the predicted score of each immune feature based on Pearson’s correlation tests, and TIDE scores were selected as core features to identify significant co-expression modules related to the immune response. Ultimately, IRRGs in significant modules (*p* < 0.05) were exported for further analysis.

### Identification of biomarkers of immunotherapy response

For co-expression modules constructed with WGCNA, hub genes of the modules of interest were identified first. For genes inside a given module, the within-module connectivity, called the module membership (MM), and the correlation with the TIDE score, called the gene significance trait (GS), were calculated as previously described [46]. Genes with high connectivity in the modules of significant immune features were identified as hub genes based on the cut-off criteria |MM| > 0.8 and |GS| > 0.2.

The expression profiles and related clinical information of PRCA samples were acquired from TCGA database. Survival analysis based on the Cox model was used to estimate the survival risk of patients under different conditions. For a specific hub gene, patients were divided into two groups according to median gene expression. Then, Kaplan–Meier curve analysis was performed with the R package “Survival” and the *p*-value between the two groups was also calculated. Hub genes (*p*-value < 0.05) that were significantly related to PRCA prognosis were considered biomarkers of the immunotherapy response.

### Construction of mRNA interaction network

To better investigate the regulatory relationships between IRRGs, we constructed an IRRG co-expression network for hub genes identified in each module of interest. Significantly correlated pairs were used to construct the network based on Pearson’s correlation coefficients. Finally, the co-expression network graphs were visualized and analyzed using Cytoscape software [47].

### Identification of candidate drugs from LINCS

To identify potential drugs to boost immunotherapy, we employed the method reported by Aissa et al. [48]. First, the mRNA expression profiles of hundreds of perturbations at various time points in PRCA cell lines were collected from LINCS. For each drug, different treatment groups, such as 6 h vs. 24 h, were compared to calculate the differentially expressed genes. According to the fold-change value associated with each gene, differentially expressed genes were ranked using Gene Set Enrichment Analysis (GSEA) [49]. Enrichment scores were calculated, and drugs with a *p*-value < 0.05 were considered significantly enriched. Specifically, a positive enrichment score shows that the drug has an upregulation effect on the target gene set, and a negative enrichment score indicates that the drug has a downregulation effect on the target gene set.

## Supplementary Materials

Supplementary Figure 1

Supplementary Table 1

Supplementary Table 2

Supplementary Table 3

Supplementary Table 4
