# Supplementary material for: Identification of biomarkers for immunotherapy response in prostate cancer and potential drugs to alleviate immunosuppression
Source: Aging (Albany NY). 2022 Jun 8;14(11):4839–57. doi: 10.18632/aging.204115 (PMC9217695; doi:10.18632/aging.204115)
Supplement: Supplementary Figure 1 [file aging-14-204115-s001.pdf]

SUPPLEMENTARY FIGURE

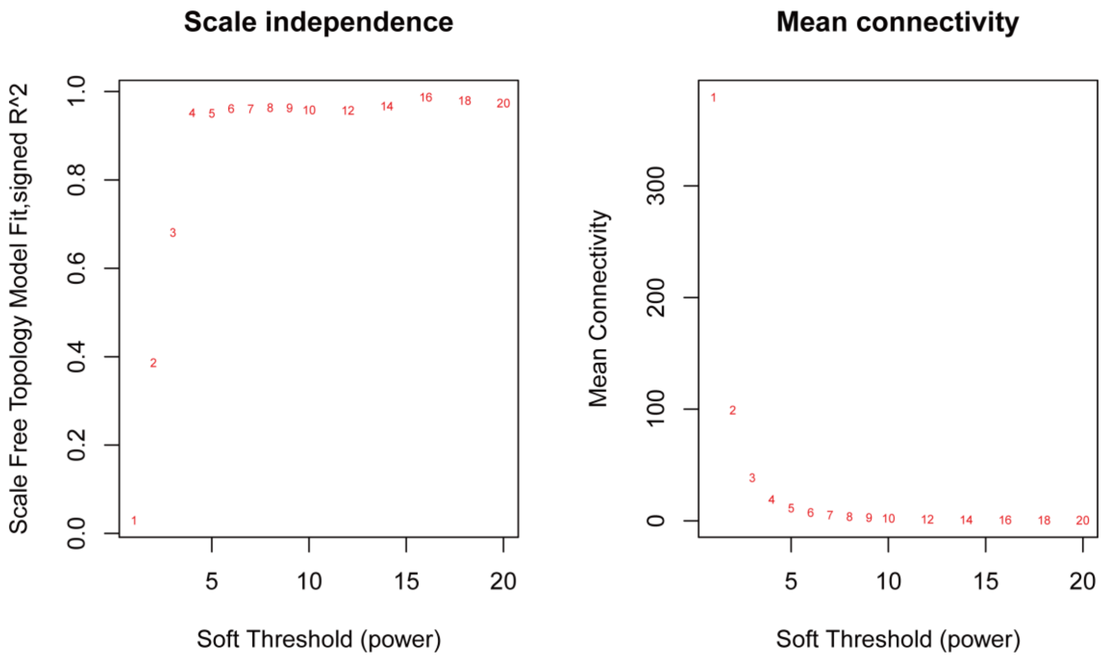

Supplementary Figure 1. Threshold determination for weighted gene co-expression network analysis.
