# Supplementary material for: Identification of biomarkers for immunotherapy response in prostate cancer and potential drugs to alleviate immunosuppression
Source: Aging (Albany NY). 2022 Jun 8;14(11):4839–57. doi: 10.18632/aging.204115 (PMC9217695; doi:10.18632/aging.204115)
Supplement: Supplementary Table 4 [file aging-14-204115-s005.pdf]

Supplementary Table 4. List of 40 of 133 hub genes expressed in LINCS and used to perform gene set enrichment analysis.

Symbol
SIX5
BAHCC1
ATN1
SNRNP70
CHPF
H1FX
INPP5E
PALM
ARFGAP1
ARHGAP33
AGPAT2
CAPN10
KIFC3
AP5Z1
ADCK2
ARHGEF1
SH3TC1
ARFRP1
C15orf39
ASMTL-AS1
ARHGEF16
CLASRP
ANAPC2
CCDC9
PPP2R3B
ALPPL2
IGFBP1
CA9
CD1A
GK2
HCRTR2
PDYN
POU4F2
SLC5A12
KRTAP5-8
CD5L
CETN1
KRT38
PDHA2
KRT84
